# Samuel Guthrie, Discoverer of Chloroform

**Published:** 1917-06

**Authors:** William V. Ewers

**Affiliations:** Physician Junior Staff Rochester General Hospital


### Samuel Guthrie, Discoverer of Chloroform.*

By DR. WILLIAM V. EWERS,

Physician Junior Staff Rochester General Hospital.

(Continued from May issue.)

Anyone wishing further data on this subject is referred to the report of the Chicago Medical Society of February 6, 1888, and the article by Professor Hubbard in the transactions of the New York Academy of Science of 1892. Professor O. P. Hubbard, Professor of Chemistry at Dartmouth from 1836 to 1883, was Professor Sillman's assistant at Yale in 1831 and received and opened the packages of chloroform that Guthrie sent to Sillman, and he tells us that he was the first to repeat Guthrie's experiments, and this before Liebig and Soubeiran had commenced theirs.

Apparently neither Guthrie nor Soubeiran realized that they had discovered a new chemical compound. They thought they were making ethyline dichloride or "Dutch Liquor," as it was called, from the Dutch chemists first making it. Chloric ether was another name for this substance. That this was so for Guthrie, is shown by the remark of Professor Sillman, in the appendix of volume XXI, page 405, where he says that "Mr. Guthrie states in his correspondence that by reading the passage in my Elements of Chemistry, Vol. II, page 20, his attention was first directed to the subject." Sillman then proceeds to quote the passage. Guthrie thought that he had discovered a cheap way of making chloric ether.

It was not until 1834 that chloroform was analyzed by Dumas and its chemical formula determined.

The chloroform that Simpson first used, which was sent him by Waldie, a Liverpool chemist, is said to have been made by Guthrie; a curious coincidence, if true. When Guthrie heard of the anesthetic properties of chloroform, he tried it on the cat so as to be competent to administer it to the soldiers if necessary.

It is the irony of fate that the son of the discoverer of chloroform should have had to undergo two operations without the benefit of anesthesia. This son was an officer in our army, serving with the troops in Mexico, and had been wounded in the knee by a guerilla. He lingered a month, and after suffering two amputations of his leg, was only released from his sufferings by death. In the letter in which he tells his daughter about the anesthetic properties of chloroform he also informs her of her brother's death.

It was thought by Guthrie and Sillman that chloroform might have some medicinal value. In order to determine this point, Guthrie sent some to Sillman who distributed several bottles among his medical friends. In the appendix to vol. XXI there is a report by one of them, Dr. Eli Ives, Professor of Theory and Practice of Medicine at Yale. All the patients except one, took the medicine by mouth; this, a pulmonic case, took it by inhalation. The most probable reason that its anesthetic property was not discovered at this time, was that the chloroform was not pure but in alcoholic solution. It was not until several months later that Guthrie produced pure chloroform. He was induced to do this by a remark of Professor Sillman at the end of vol. XXI, where he asks if a method cannot be devised to detach the alcohol from the chloric ether "and obtain the latter concentrated and in quantity."

Dr. Ives' report is dated January 2, 1832.

There are several other articles in vol. XXI by Guthrie dealing with chemical experiments and methods of manufacture. In vol. XXII he has an article on the production of pure chloric ether. One of his interesting experiments though not recorded in this journal, is with the French sugar beet, which he raised one summer. The crop of beets was large but the quantity of sugar obtained small. This ended his experiments in sugar beet raising.

With the exception of volume one, I have searched all the volumes of the American Journal of Science and Arts up to the time of Guthrie's death to see if there were any other articles by him; unfortunately none could be found. It seems strange that his contributions to science should be limited to this short space of time. Whether he wrote for other journals I do not know. The only explanation I can see for this is, that at this time his sons had reached an age where they could relieve him of many of the cares of his manufacturing interests, enabling him to devote more time to his laboratory. Later one of them moved west and I presume the other became so engrossed with his own affairs that he could not help his father to any great extent.

He is said to have had a secret process for manufacturing alcohol that produced a very fine product. This secret he never revealed, so we are unable to judge of its value. Though the most important part of biography is to record what a person has accomplished, to me that which tells of the man himself is far more interesting. Fortunately for me there are still persons living who knew Dr. Guthrie, so we are able to have a better insight into his character. Unfortunately for us there is no picture of the Doctor in existence.

He only had one taken. The numerous explosions through which he had passed had not improved his looks, and besides he had the tic douloureaux. When the Doctor saw his picture he destroyed it, saying if he was as damn homely as that he did not want his descendants to see it.

He was a rather quiet man, making frequent use of the words yes and no. Though taciturn with strangers he was free with his friends. That he was liberal, at least with his family, his letters show. In most of them he mentions enclosing ten, twenty or more dollars. He had quite a library for those days, though books on chemistry and encyclopedias are said to predominate. Still works of fiction were present. He considered that the library was for the use of the family, and there were no restrictions, even on the children, as to what they should read. His granddaughter says that the only rule she remembers the Doctor was particular about was that no one should turn down the leaves of the books.

In regard to children, we are told that he did not believe in the use of the rod as a means of discipline. His descendants point with pride to the fact that in spite of such views, his children all turned out well. Dr. Guthrie was always interested in the progress that was made in science and the arts. Knowing his interest in such things, the villagers of Sackett's, on his return from New York, always expected a description of anything new or wonderful that he had seen. His grandson, Ossian Guthrie, states that he well remembers his telling of the discovery of Daguerre; the Morse telegraph, and the year-running clock. On the trip when he saw the latter he was accompanied by a friend, a Mr. Harlow, who possessed great mechanical skill. A few days after their return, Mr. Harlow produced one of these clocks, which he had made, to the delight of the village.

The Doctor gradually gave up the practice of medicine, and during the latter years of his life practiced very little, though he would take a case now and then. There is a story that a certain young lady, who had an arm that was either diseased or injured, which, tradition does not say, was advised by different doctors to have it amputated. Her parents finally took her to Doctor Guthrie, who saved the arm. Tradition does not say if the grateful patient paid the Doctor. The Doctor was evidently fond of his little joke, for when he saw the batteries that were used in connection with the telegraph, he made one, which he used to shock the villagers.

That his sense of humor did not desert him even when he was the victim, the following seems to show. On one occasion he placed some powder under the stove to dry and then went to bed. During the night the powder exploded. Fortunately the doors opened outward so no great damage was done. On being thus rudely awakened the Doctor's only remark was: "that was a damn Guthrie trick."

In his later years he had to face adversity. Sackett's depended for its prosperity upon its importance as a lake port as well as its proximity to the garrison. The railroad was now pushing its way into the north country, and commerce turned from the lake route to the new channel. This of course affected Sackett's adversely, and undoubtedly contributed to the decline of the fortunes of the Doctor and his sons. The son who died in Mexico left his affairs in bad shape, and the other one failed for 50,000 dollars, a large sum for those days. The Doctor evidently faced the situation philosophically, for in his letters there is no complaining. Instead he took a hopeful view of life, and made plans for his future activities.

It was in this frame of mind that he died, October 19, 1848.

Such was the man who lived and worked in one of the remote parts of our state nearly a hundred years ago. The house where he lived is still standing, and it seems that it would be fitting, because of his discovery of chloroform, if at some time our state society would mark the place with a memorial tablet.

I wish to extend my thanks to those who have so kindly helped me in the preparation of this paper. Especially am I indebted to Mr. T. S. Chamberlain of Chicago, grandson of Dr. Guthrie, for his indispensible help in collecting much of the data. I also wish to thank Mr. Pettit of Sackett's Harbor for his kindly advice.

---

**Fracture of Trapezoid.** A. Mouchet, Presse Med., Oct. 9, 1916, reports a case in a female weaver, aged 30, who was suddenly incapacitated while lifting a heavy bolt of cloth with the hand strongly flexed (50 kilos, with assistant lifting at each end of the bolt). A transverse fissure of the trapezoid alone was found radiographically, with a sort of dorsal eversion of the fragments. Warm bathing and a retentive dressing with a soft wad, resulted in healing in 3 weeks, but with an osseous projection on the dorsal aspect of the trapezoid. He considers isolated fracture of this bone as unique.

---

**Apparent Case-to-Case Pneumonic Infection,** wife to husband, is reported by F. S. Lister in the Med. Jour. of S. Africa. Strain G. was isolated and demonstrated serologically—this being rare in experience, C having been found in all of 9 recent cases in Europeans.

